# A double-masked placebo-controlled trial of azithromycin to prevent child mortality in Burkina Faso, West Africa: Community Health with Azithromycin Trial (CHAT) study protocol

**DOI:** 10.1186/s13063-019-3855-9

**Published:** 2019-12-04

**Authors:** Ali Sié, Mamadou Ouattara, Mamadou Bountogo, Cheik Bagagnan, Boubacar Coulibaly, Valentin Boudo, Elodie Lebas, Jessica M. Brogdon, Ying Lin, Till Bärnighausen, Travis C. Porco, Thuy Doan, Thomas M. Lietman, Catherine E. Oldenburg, for the Étude CHAT Study Group

**Affiliations:** 1 Centre de Recherche en Santé de Nouna, Nouna, Burkina Faso; 2 Francis I. Proctor Foundation, University of California, 513 Parnassus Ave, Room S334, San Francisco, CA USA; 3 Heidelberg Institute of Global Health (HIGH), Heidelberg, Germany; 4 Africa Health Research Institute (AHRI), Somkhele, South Africa; 5 Department of Global Health and Population, Harvard T.H. Chan School of Public Health, Boston, MA USA; 6 Department of Ophthalmology, University of California, San Francisco, CA USA; 7 Department of Epidemiology and Biostatistics, University of California, San Francisco, CA USA

## Abstract

**Background:**

Biannual, mass azithromycin distribution has previously been shown to reduce all-cause child mortality in sub-Saharan Africa. Subgroup analysis suggested that the strongest effects were in the youngest children, leading to the hypothesis that targeting younger age groups might be an effective strategy to prevent mortality. We present the methods of two randomized controlled trials designed to evaluate mass and targeted azithromycin distribution for the prevention of child mortality in Burkina Faso, West Africa.

**Methods/design:**

The Child Health with Azithromycin Treatment (CHAT) study consists of two nested, randomized controlled trials. In the first, communities are randomized in a 1:1 fashion to biannual, mass azithromycin distribution or placebo. The primary outcome is under-5 all-cause mortality measured at the community level. In the second, children attending primary healthcare facilities during the first 5–12 weeks of life for a healthy child visit (e.g., for vaccination) are randomized in a 1:1 fashion to a single orally administered dose of azithromycin or placebo. The primary outcome is all-cause mortality measured at 6 months of age. The trial commenced enrollment in August 2019.

**Discussion:**

This study is expected to provide evidence on two health systems delivery approaches (mass and targeted treatment) for azithromycin to prevent all-cause child mortality. The results will inform global and national policies related to azithromycin for the prevention of child mortality.

**Trial registration:**

ClinicalTrials.gov, ID: NCT03676764. Registered on 19 September 2018; prospectively registered pre results.

## Background

Despite a secular trend of decreasing under-5 mortality rate (U5MR) across sub-Saharan Africa, childhood mortality remains persistently high in some regions [1]. The development of innovative and scalable approaches for reducing U5MR in high-burden regions is a priority. The MORDOR study recently showed that biannual, mass azithromycin distribution reduces all-cause child mortality by 14% compared to placebo in districts in Niger, Tanzania, and Malawi [2]. By far the largest effects were seen in Niger, which had the highest U5MR of the three study sites, with nearly one in five deaths averted. Mass drug administration with azithromycin has been used by trachoma control programs for decades [3] and is highly effective at clearing the ocular strains of *Chlamydia trachomatis* that cause trachoma [4, 5]. Mass azithromycin distribution for trachoma control has also been shown to reduce child mortality in some settings [6, 7].

In the MORDOR study, the largest effects of azithromycin treatment were in children aged 1–5 months old, with a nearly 25% reduction in mortality in this subgroup. MORDOR was a large simple trial [8], and was not powered to detect differences in subgroup by age. However, this result generated the hypothesis that treatment of the youngest children may yield the greatest benefit for prevention of mortality. Biannual mass distribution of azithromycin is not efficient for reliably treating the youngest children, as, on average, children under 6 months of age in any given treatment phase are reached at approximately 4 months of age. If treatment of the youngest children proves to be the most effective strategy for reducing mortality with azithromycin, alternative delivery mechanisms to mass distribution will need to be identified.

Here, we describe two nested, randomized controlled trials in which we evaluate the efficacy of targeted azithromycin distribution in both the presence and the absence of biannual mass azithromycin distribution using a hierarchical factorial design [9]. We evaluate the effect of azithromycin administered to infants who are attending healthy child clinic visits during their fifth through to their twelfth week of life, compared to placebo on infant mortality rates. These trials seek to determine (1) the efficacy of azithromycin compared to placebo administered to young infants for the prevention of infant mortality and (2) the efficacy of biannual mass azithromycin distribution to children aged 1–59 months compared to placebo for reducing all-cause child mortality.

## Methods/design

### Study design

**Fig. 1:**
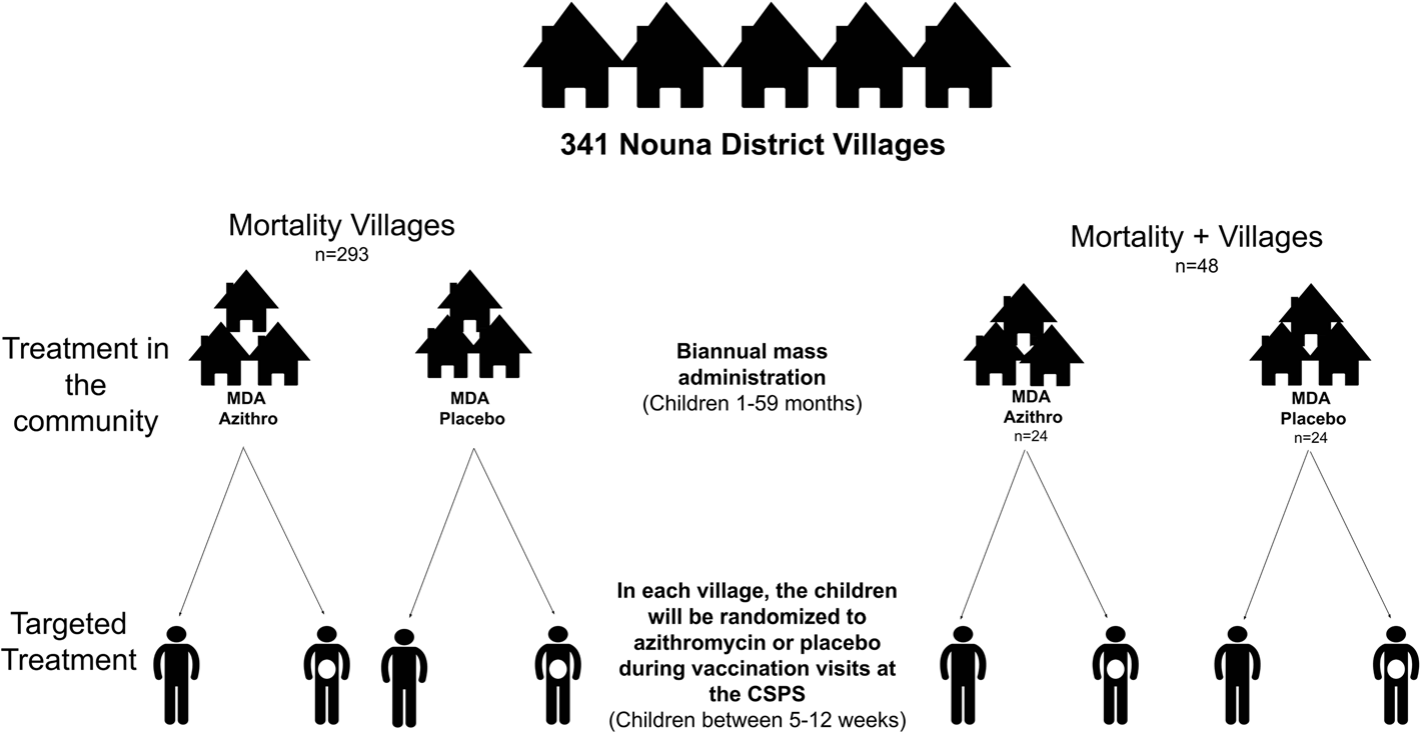
Study flow diagram for the Child Health with Azithromycin Treatment (CHAT) study in Nouna District, Burkina Faso, West Africa. This study is a hierarchical factorial study in which communities are randomized to biannual, mass, single-dose azithromycin or placebo distribution to all children aged 1–59 months and infants aged 5–12 weeks are individually randomized to a single dose of azithromycin or placebo. Abbreviations: *MDA* mass drug administration, *CSPS Centre de Santé et de Promotion Sociale* (primary healthcare facility)

The Child Health with Azithromycin Treatment (CHAT) study consists of two double-masked placebo-controlled, nested, randomized trials (Fig. 1). In the first trial, communities are randomized in a 1:1 fashion to biannual, directly observed, orally administered, mass azithromycin or biannual placebo distribution to children aged 1 to 59 months. In the second trial, individuals born into the communities who are between 5 and 12 weeks of age are randomized in a 1:1 fashion to a single dose of directly observed, orally administered azithromycin or placebo during a healthy child visit at a participating Centre de Santé et de Promotion Sociale (CSPS). These are nurse-led primary healthcare facilities that provide basic preventative and treatment services, including antenatal care, postnatal care, and vaccination. The trial commenced enrollment in August 2019 and recruitment is expected to last until late 2022. This protocol is reported according to the Standard Protocol Items: Recommendations for Interventional Trials (SPIRIT) guidelines.

### Objective and hypothesis

The objectives of this study are (1) to determine the efficacy of biannual mass azithromycin distribution to children aged 1–59 months on all-cause under-5 mortality and (2) to determine the efficacy of a single targeted dose of azithromycin administered during well-child visits to children aged 5 to 12 weeks of age on 6-month mortality. We hypothesize that biannual mass azithromycin distribution will reduce all-cause child mortality relative to placebo and that a single targeted dose of azithromycin will reduce all-cause infant mortality relative to placebo.

### Study oversight

An independent Data and Safety Monitoring Committee (DSMC) oversees the data collected in this study. The DSMC contains members with expertise in pediatrics, infectious disease, biostatistics, ethics, and mass azithromycin distribution. The DSMC meets annually in a face-to-face meeting, once before the study began and then for ongoing monitoring annually. Quarterly phone meetings are conducted to monitor progress of the trial, as well as ad hoc meetings as needed. Study investigators conduct monitoring visits to the study site at least biannually. The principal investigators will notify the DSMC, sponsors, and institutional review boards of any changes in the study protocol and then will communicate changes to study sites. Any deviations from trial protocols are documented and reported as necessary.

### Setting

**Fig. 2:**
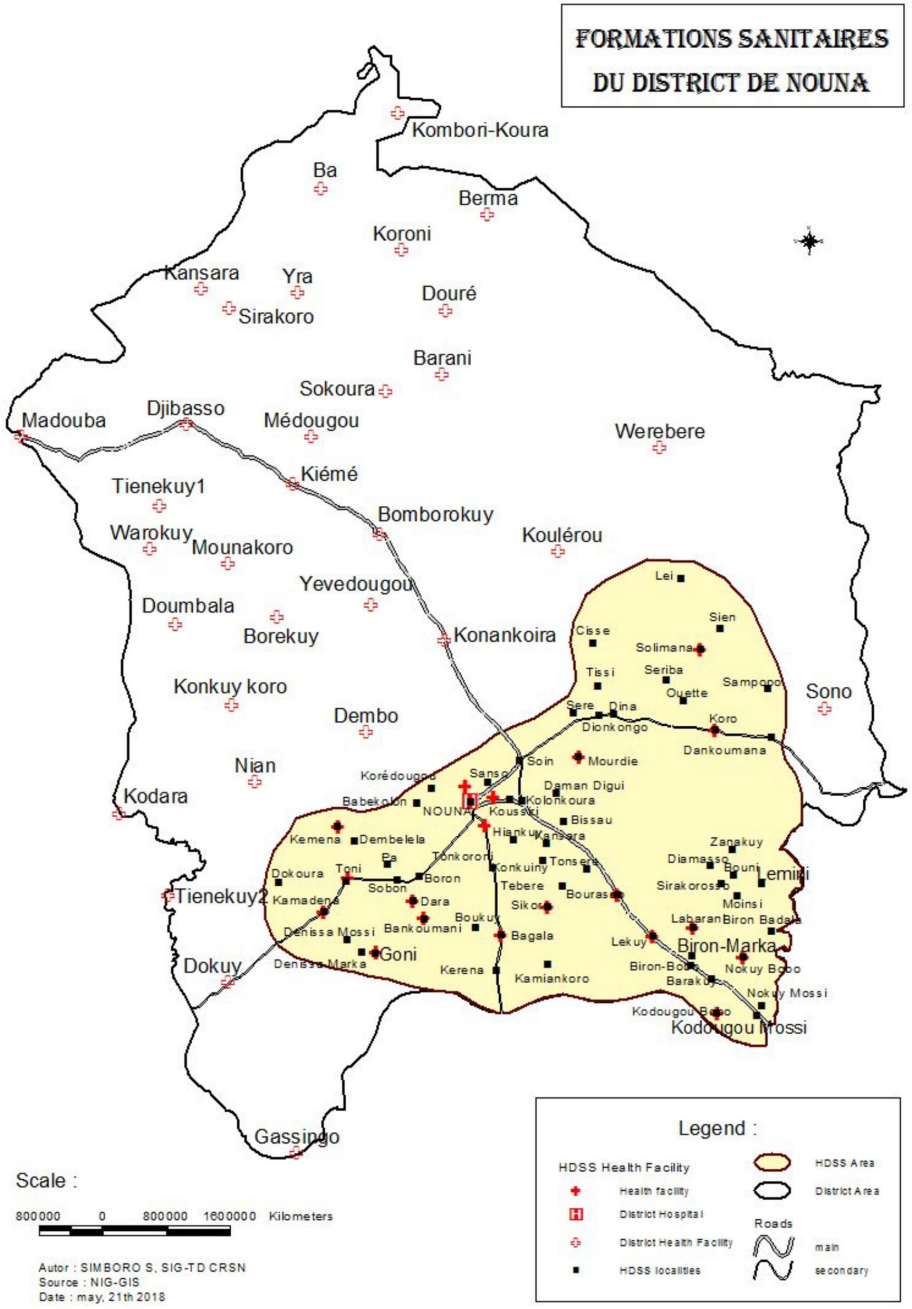
Study area in Nouna District, Burkina Faso, West Africa. The shaded area indicates the Nouna Health and Demographic Surveillance site area of Nouna District. Primary health facilities are indicated with red crosses. Abbreviations: *HDSS* Health and Demographic Surveillance Site, *NIG-GIS* geographic information system

This study is taking place in Nouna District in the Boucle du Mouhoun region of northwest Burkina Faso (Fig. 2). Burkina Faso is a landlocked country located in francophone West Africa. The trial covers the area of both the Nouna Health and Demographic Surveillance Site (HDSS) [10], which covers approximately one third of Nouna District, as well as the non-HDSS area. The area is rural and agrarian, and most inhabitants are farmers and cattle keepers [11]. The U5MR in Boucle du Mouhoun was estimated at 110.8 per 1000 live births in 2015 [1].

### Study development and community engagement

This study was developed in consultation with local, national, and global policy-makers and stakeholders in child health and survival. Community sensitization related to the study was done with leaders in each study community. Local healthcare providers at each CSPS were engaged prior to the start of the study, are kept informed about study progress, and are involved in informing potential participants about the study and facilitating follow-up.

### Community recruitment

From November 2018 through March 2019, all communities in Nouna District were mapped. All communities located in the district received permission from local leaders to participate and have been included in the study.

### Individual recruitment

Children aged 28 days of age and older are recruited for the study through well-child visits at the CSPSs in Nouna District. Recruitment visits include village-based recruitment days, vaccination visits (e.g., during the first Expanded Program on Immunization visit which typically occurs between 6 and 8 weeks), postnatal care visits (in the study area, a postnatal visit typically occurs at approximately 42 days of life), or any other well-child visit that occurs during the early infancy period. To target treatment as early as possible when the risk of mortality is highest, we will attempt to enroll children under 56 days of age. Children will be eligible for inclusion in the study if aged up to 84 days of life (12 weeks) at enrollment.

### Inclusion and exclusion criteria: communities

All communities in Nouna District with the exception of Nouna Town are eligible for inclusion in the study, regardless of size. Nouna Town is excluded because it is a semi-urban community with lower mortality and greater access to healthcare, and thus not comparable to the rural communities that make up the remainder of the district. To be eligible for the study, the community leader must provide their consent for the community to participate.

### Inclusion and exclusion criteria: individuals

Due to the risk of infantile hypertrophic pyloric stenosis (IHPS), children under 28 days of age are excluded from the study [12]. Observational evidence has suggested that macrolide use during the neonatal period may increase the risk of IHPS [12, 13]. The safety and efficacy of macrolide use during the neonatal period for the prevention of infant mortality is currently being tested in a concurrent randomized controlled trial in Burkina Faso [14].

### Randomization

The randomization list was developed by TCP and YL in R version 3.5.1. Community randomization is stratified by communities inside and outside the HDSS. Treatment letters were randomly assigned to represent azithromycin or placebo, and communities were randomly assigned to a treatment letter using a masked random seed value [15]. For individuals, study identification numbers were assigned to a treatment letter (representing azithromycin or placebo) and pre-loaded into the study application. During enrollment, the study application displays the treatment letter associated with the child’s identification number once the child is enrolled in the study and the identification number has been assigned.

### Census

**Fig. 3:**
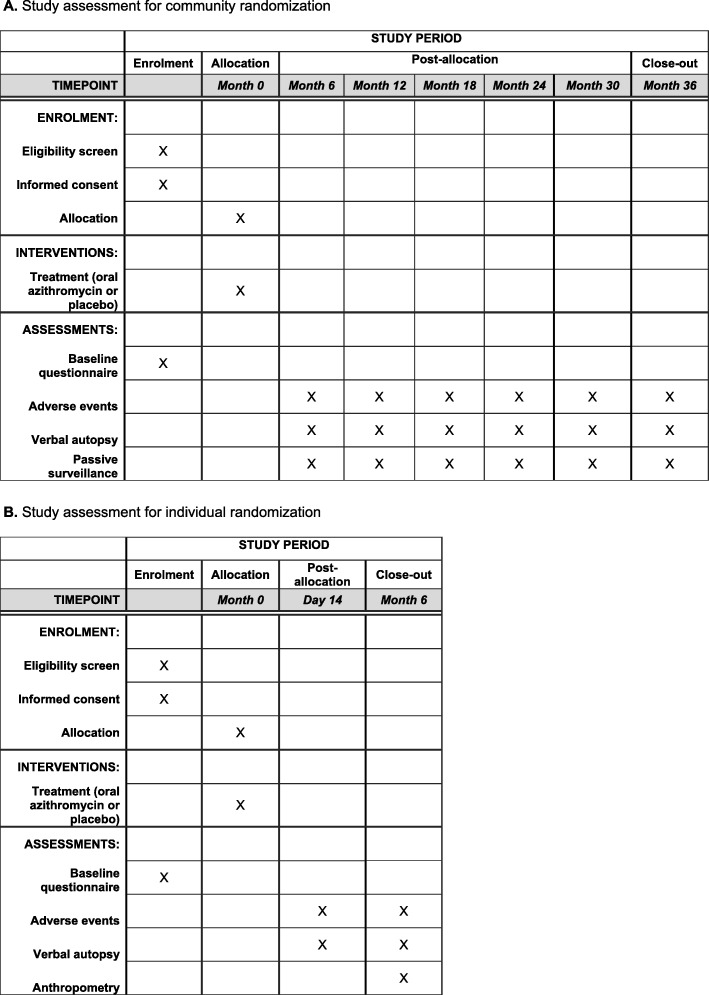
Study assessments for community randomization (**a**) and individual randomization (**b**)

In all study communities, a door-to-door enumerative census is conducted every 6 months (Fig. 3a). The census focuses specifically on children aged under 5 years who are living in the household. For each child, information is collected on their vital status (died, alive, or moved). The census is conducted prior to treatment.

### Community-based treatment and masking

Following each biannual census, children aged 1–59 months are treated with a single, directly observed 20-mg/kg dose of orally administered azithromycin as a pediatric suspension, identical to that used in trachoma control programs [3, 16]. Those who are able to stand are measuring using a height stick, identical to that used in the trachoma control program in Burkina Faso, and dosed accordingly. Children who are too young to stand are weighed using a hanging scale. During randomization, each community was randomly assigned a treatment letter that corresponds to either azithromycin or placebo. Each community is treated with bottles labeled with the corresponding treatment letter. With the exception of the treatment letter on the label, the azithromycin and placebo and their labels and bottles are identical in appearance. Participants, treatment workers, outcome assessors, and investigators are masked to community treatment assignment.

### Individual treatment and masking

Children recruited and enrolled in the individual study will be treated with a single, directly observed, orally administered, 20-mg/kg dose of azithromycin. After enrollment in the study, each child is assigned a treatment letter that will correspond to either azithromycin or placebo. Treatment bottles are marked with treatment letters but are otherwise identical in appearance. Participants, study staff, and investigators are masked to individual treatment assignment.

### Data collection and management

Electronic data are captured in the field using the SurveySolutions platform (World Bank Group, Washington, DC, USA).

### Individual follow-up

Infants enrolled in the individual treatment trial will be followed at 180 days (Fig. 3b). An additional visit at 14 days is conducted in a random sample of 10% of children for adverse event screening. The primary outcome visit is the 180-day visit. The location of residence and at least one phone number (if available) are collected for the caregiver of each child enrolled in the study. Caregivers of study participants receive follow-up reminders from study personnel, and if they do not attend a follow-up visit in the healthcare facility, a home visit is attempted.

### Primary outcome measurement: community

The primary outcome of the community-randomized portion of the study is all-cause mortality per the census. To count towards the primary outcome, children must be counted on one census and recorded as having died in a subsequent census.

### Primary outcome measurement: individual

The primary outcome for individually randomized children is mortality at the 180-day visit. Mortality is measured via vital status assessment (categorized as alive, died, moved, or unknown).

### Secondary outcome measures: community

In a subset of 48 communities within the HDSS (“mortality-plus communities”), samples are taken from a population-based random sample of 15 children aged 1–59 months per community at 0 and 36 months. Samples include rectal swabs for the assessment of the intestinal microbiome [17–19], nasopharyngeal swabs for the assessment of macrolide resistance in commensal pneumococcus, dried-blood-spot collection for assessment of serologic markers of exposure to pathogens, and thin and thick smears for malaria.

### Secondary outcome measures: individual

Anthropometric measurements are recorded for all individuals at baseline and the 180-day visit, including weight, height, and mid-upper-arm circumference.

### Passive surveillance

Passive surveillance is undertaken at all CSPSs in the study catchment area. During the study period, healthcare visits including reasons for the visit, diagnosis, and treatment plan are recorded for children under 59 months of age. These data are linked to the community and to individual children via recording the community in which the child resides and the child’s study identification number in the electronic data collection application. Participation in the trial will not require alteration to usual care pathways or use of any medications should children need care during the course of the trial. Usual care will continue for both arms of the trial, and because the study is masked, we do not anticipate differential access to care in children receiving azithromycin compared to placebo. Because the trial is enrolling healthy participants, there are no anticipated harms or provisions for post-trial care.

### Adverse events

An adverse event survey is conducted among all children aged 1–5 months in the 48 mortality-plus communities approximately 2 weeks following treatment. Similarly, caregivers of a random sample of 10% of individually randomized children will be interviewed for adverse events 2 weeks after treatment. Previous evidence from MORDOR showed no evidence of a difference in safety events in infants receiving azithromycin compared to placebo, and the most common adverse events were gastrointestinal, with a decrease in caregiver-reported diarrhea in children receiving azithromycin compared to placebo [20]. As in MORDOR, adverse event screening in CHAT is designed for detection of a safety signal rather than to identify every adverse event in the study population due to the scale of the study and excellent safety profile of azithromycin for children in MORDOR and trachoma control settings [21, 22].

### Interim analysis: community

A single pre-specified interim analysis for efficacy will be conducted when mortality data are available for the first year (two study phases). The interim analysis will use an alpha of 0.001, and the final analysis will be conducted at an alpha of 0.049.

### Interim analysis: individual

A single pre-specified interim analysis for efficacy will be conducted when mortality data are available for one full year of recruitment (e.g., 18 months after the start of the trial). The interim analysis will use an alpha of 0.001, and the final analysis will be conducted at alpha 0.049.

### Statistical analysis

The primary analyses for community randomization and individual randomization are pre-specified to be conducted separately, with a secondary analysis planned to evaluate any interaction due to the factorial design of the trial. Mortality is a rare event, and the study is not powered to detect an interaction between the two interventions [8]. We note that the existence of the two co-interventions is an external, not an internal, validity concern, and results will be interpreted in the context of the other azithromycin intervention. All analyses will be conducted as intention-to-treat.

### Statistical analysis: community

The primary analysis will be conducted as negative binomial regression. The number of person-years for persons in the target age range will be derived by summing the person-time for each study phase. The community-level predictor will be treatment arm of the community. Due to the cluster randomized nature of the study, primary analyses for efficacy will be conducted at the cluster level. Statistical significance will be assessed by permutation testing at the level of the randomization unit, with a *P* value threshold for statistical significance of 0.049 (see “Interim analysis”).

### Statistical analysis: individual

The primary analysis will be conducted as a binomial regression with a complementary log-log link. This model estimates the relative hazard of mortality in children receiving azithromycin compared to placebo and allows for varying person-time to be contributed by different individuals. The analysis will be conducted at the level of the individual. Statistical significance will be assessed by permutation test, with a *P* value threshold for statistical significance of 0.049 (see “Interim analysis”).

### Statistical analysis: factorial design

As a pre-specified secondary analysis, we will assess interaction between the community and individual azithromycin interventions. The factorial analysis will be conducted at the individual level using a binomial regression model with a complementary log-log link, with standard errors adjusted for clustering at the level of the community. The model with include terms for the individual and community randomization and the interaction term for the two interventions.

### Sample size considerations: community randomization

The sample size calculation for the community trial is based on the primary outcome, all-cause mortality as measured by the census and is calculated based on the number of randomization units (communities) in the study area (*N* = 341), which was determined during the pre-study mapping. We assume a rate of 20 deaths per 1000 person-years in children aged 1–59 months, based on previous studies in Niger [2] and data from the Nouna HDSS [10]. The coefficient of variation was estimated from the MORDOR-Niger study as 0.34, that the average community size was 1000 people, and that 16.7% of the population is in the target age rage (1–59 months). Under these assumptions the inclusion of 341 clusters (170 and 171 per arm) would yield at least 80% power to detect a difference in mortality of 13.65% with a two-sided alpha of 0.05. We anticipate that approximately 450,000 treatments will be administered over the 36-month study.

### Sample size considerations: individual randomization

The sample size calculation for the individual trial is based on the primary outcome, all-cause mortality at 6 months of age in individuals. We assume that mortality from 1 to 6 months is 40 deaths per 1000 live births and loss to follow-up will be 5%. A sample size of 32,702 infants (16,351 per arm) would provide at least 80% power to detect a 15% difference in the probability of mortality.

### Dissemination plan

The results of this study will be presented and local, national, and international meetings. The results will be presented to key stakeholders in Burkina Faso and elsewhere, including to the Ministry of Health in Burkina Faso. The results will also be published in an open-access format in peer-reviewed journals. Primary analyses for community and individual randomization will be published separately.

## Discussion

The MORDOR study demonstrated that biannual mass azithromycin distribution reduces all-cause child mortality in some settings [2, 23]. This study led to considering the inclusion of mass drug administration with azithromycin for child mortality in World Health Organization guidelines in regions with very high child mortality. The mechanism behind this effect on mortality is unknown, although presumably is via a reduction of the infectious burden [24]. Although evidence of child mortality in trachoma programs generally corroborates the findings in MORDOR [6, 7, 25], when and where azithromycin will be effective for reducing child mortality remains unknown. MORDOR was a large simple trial with a rare outcome (mortality), and thus the detection of effect modification by geography was difficult [26, 27]. Although there was no significant difference in mortality rates by study country, the study was not specifically designed to detect differences between countries. The CHAT study will provide evidence of the efficacy of biannual mass drug administration with azithromycin for the prevention of child mortality in a different time and place than MORDOR, but in a region with child mortality rates that are nearly as high as those in the Niger site of MORDOR.

Although subgroup analysis of MORDOR suggested that the greatest effects were in younger (1–5-month-old) children, there was no significant difference in the efficacy across subgroups. This finding led to the hypothesis that targeting younger children may be an efficacious approach to preventing mortality. This study investigates an alternative health system delivery approach – distribution of azithromycin during early well-child visits that are well established in the health system. We anticipate that this study will provide evidence both of the efficacy of targeting azithromycin to young infants as well as the efficiency of integrating azithromycin into child health visits at healthcare facilities. Several other ongoing studies are currently investigating applications of azithromycin for child health. For example, the ABCD study (ClinicalTrials.gov, ID: NCT03130114) is a multisite, individually randomized study investigating the efficacy of azithromycin compared to placebo on 6-month mortality in children under 2 years of age who are recovering from an acute diarrheal episode. The Toto Bora trial (ClinicalTrials.gov NCT02414399) is evaluating the efficacy of a 5-day course of azithromycin on post-discharge mortality in children aged 1–59 months who had been previously hospitalized [28]. The PregnAnZI-2 study (ClinicalTrials.gov NCT03199547) is evaluating the efficacy of maternal azithromycin administration on neonatal sepsis. The results of the CHAT study will, therefore, become available in the context of other studies that are testing azithromycin in similar applications. Taken together, we anticipate that these studies will yield a wealth of evidence related to the role of azithromycin for child health via multiple potential delivery pathways and health conditions.

Evidence from trachoma control studies has generally shown that mass azithromycin distribution for trachoma control selects for macrolide resistance in some organisms [29]. In MORDOR, communities receiving azithromycin had a higher prevalence of macrolide resistance in pneumococcal isolates and also showed evidence of expansion of the resistome [30]. Through the collection of nasopharyngeal and rectal samples in the mortality-plus communities of CHAT, we will build on this work by evaluating antimicrobial resistance in macrolides and other antibiotic classes in communities receiving azithromycin and placebo for 36 months. Given that azithromycin will be given to approximately half of young infants in the study communities, antimicrobial resistance data from the placebo communities will provide some evidence of macrolide resistance in communities where children are being treated at an individual level.

There are several limitations to the design of this study to consider. First, the scale of a study required to evaluate a rare endpoint, such as mortality, is very large. The large, logistically complicated scale of the studies requires that the study be designed and conducted as a “large simple trial” [8]. These trials improve efficiency by reducing the complexity of a trial’s design. Large simple trials have very simple inclusion criteria and simple outcome assessment methods, often measuring few secondary endpoints. While such steps are necessary to achieve the sample size required for the trial to be adequately powered, the number of additional outcomes measured is limited. The multilevel factorial design of the study means that there are no communities in which some azithromycin is not being distributed (e.g., placebo communities will have children receiving azithromycin during well-child visits). While this allows for assessment of the health facility intervention in the presence of community azithromycin versus placebo, there is no pure comparison group. Additional future planned studies will evaluate mass distribution of azithromycin in settings other than MORDOR and include pure placebo communities. This study will answer similar, but subtly different, research questions about azithromycin implementation for child mortality.

## Conclusions

The results of this study are expected to provide evidence of the efficacy of mass and targeted azithromycin distribution for the prevention of child mortality. This will inform policy about different health system approaches for delivering azithromycin, as well as providing evidence of its efficacy in a different population several years after completion of the first MORDOR study. If proven to be effective, the results of this study could lead to policies recommending integration of azithromycin in primary healthcare facilities, either as a standalone intervention or in conjunction with mass azithromycin distribution.

## Trial status

This protocol is version 2.9, 7 May 2019. Recruitment began on 1 August 2019 and is expected to last until approximately 31 July 2022.

## Supplementary information

**Additional file 1.** Standard Protocol Items: Recommendations for Interventional Trials (SPIRIT) 2013 Checklist: recommended items to address in a clinical trial protocol and related documents*.

**Additional file 2.** Sample informed consent documents.

## Data Availability

Not applicable. Upon completion of the trial, de-identified data will be made publicly available per the Bill and Melinda Gates Foundation policy.

## Abbreviations

CHAT: Community Health with Azithromycin Treatment
CSPS: Centre de Santé et de Promotion Sociale
DSMC: Data and Safety Monitoring Committee
HDSS: Health and Demographic Surveillance Site
IHPS: Infantile hypertrophic pyloric stenosis
U5MR: Under-5 mortality rate
