# Supplementary material for: A double-masked placebo-controlled trial of azithromycin to prevent child mortality in Burkina Faso, West Africa: Community Health with Azithromycin Trial (CHAT) study protocol
Source: Trials. 2019 Dec 4;20:675. doi: 10.1186/s13063-019-3855-9 (PMC6894235; doi:10.1186/s13063-019-3855-9)
Supplement: Supplementary file 2 — Additional file 2. Sample informed consent documents. [file 13063_2019_3855_MOESM2_ESM.zip › signed consent-parental-vaxvisit-3.0 May 2019R1.docx]

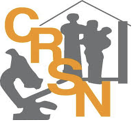

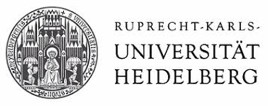

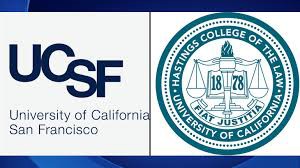


**Parental or Legal Guardian Consent for Participation in the Study**

**Information Notice:**

For parents or legal guardians of children under 12 weeks of age attending visit at the CSPS Title: Utilization of Azithromycin for the Reduction of Mortality in Young Children

Date: 5/22/2019

Version: 3.2

**Significance of Consent**:

“Consent” signifies that you authorize your child to participate in this clinical research study. You have the right to decide if you want your child to participate or not. The objective of this document is to explain the study to you. Please take the time to read or listen attentively to the following information.

This is a medical research study. This study will be explained to you by a staff member from the Centre de Recherche en Santé de Nouna (CRSN). The principal investigator, Dr. Ali Sié from the CRSN, tel: +22670252957 and the other principal investigator for this study, Dr Thomas Lietman from the university of California in San Francisco, are available by phone to answer your questions at +1 415-502-2662.

This medical research only includes the people who have chosen to participate. Take your time in making your decision to participate in this study. You can discuss your decision with your friends and family. If you have questions, you can ask the study physicians or the CRSN personnel. We ask for the participation of your child that is under 12 weeks old because your child lives in a village that is part of the study. You could choose to participate in this study or not. The participation of your child is voluntary. If you accept that your child participates, you will receive a copy of this signed document.

**Why is this study being conducted?**

The objective of this study to look at how the distribution of antibiotics affect childhood mortality.

**What is the role of CRSN?**

The Center of Health Research in Nouna is one of four research institutes in the ministry of health. It has functioned as a demographic health surveillance system since 1992. The DHSS of Nouna is located in the health district of Nouna in northwest Burkina Faso, about 300 kilometers from the capital, Ouagadougou. The CRSN explores various subjects depending on national and global research priorities. The CRSN has more than 20 years of professional experience within the communities of the Nouna region.

**What is the role of the University of San Francisco?**

UCSF is a public research university in San Francisco, California. For this project, UCSF will oversee all study related activities. UCSF investigators will design, analyze, and provide oversight for this research in close collaboration with the CRSN. In addition, UCSF will coordinate monitoring, training, and evaluation visits throughout the duration of the study.

**How many children/households will participate in this study?**

Approximately 15,000 children per year for a total of 3 years will participate in this study.

**What happens if your child participates in the study?**

If you decide to participate in this study, we will:

- Treat your child with a single dose of azithromycin or placebo while you are visiting the CSPS:
  - The allocation of Azithromycin or placebo is randomized randomly
  - The treatment is a liquid solution that your child can swallow.
  - Your child could receive the antibiotic, or they could receive the placebo. A computer program will randomly decide which group your child will be in, like a lottery. We cannot know or decide which group your child will be in.
  - The placebo will look and taste the same as the antibiotic, but it will not contain any active substance.
- Measure your child's height, weight and mid-upper arm circumference to make sure your child is not at risk for malnutrition.
- When your child is approximatively 6 months old we will ask questions about your child’s health and we will measure your child’s height, weight and mid-upper arm circumference when you are visiting the CSPS.

**How long will my child be in the study?**

You can expect your child to be in the study for a total of 30 minutes.

The anthropometry assessment for the baseline and 6 month visit will take a total of 5 minutes each. The treatment at baseline will take approximately 5 minutes. Your child can be selected for a 2-week secondary effects assessment. If your child is selected, the assessment will take about 10 minutes. The vital status questionnaire at 6 months will take about 5 minutes.

**Can my child stop participating in the study?**

Yes. You can decide to stop participating at any moment. Inform the study physician or the CRSN personnel if you are considering stopping or if you decide to stop. They will tell you how to stop your child’s participation safely.

**What are the secondary effects or the risks my children can expect to suffer?**

Your child can have secondary effects during the study. The secondary effects frequently associated with this type of antibiotic are: diarrhea, abdominal pain, vomiting, or skin rashes. Each child participating in this study will be carefully monitored for any side effects.

Your child may also be allergic to the study medication, the most common allergic reactions to this type of treatment are: a rash, itching or dizziness. Serious allergic reactions such as shortness of breath or swelling of the face / tongue / lips are rare and will be closely monitored and treated free of charge by our team.

The antibiotic treatment can change your child’s bacterial diversity, but this change will only be temporary and the diversity of bacteria living in your child's intestine and nose will return to normal after a few weeks after treatment.

**What are the advantages of participating in the study?**

If your child is in the group receiving antibiotics and suffers from an undiagnosed infectious disease, they could directly benefit from receiving this antibiotic.

**What other choices do I have if my child is not participating in this study?**

You can decide to participate in the study or not to participate in the study. Your participation is voluntary. It is your choice if your child participates in the study or not. Nothing will happen to you or your child if you decide that your child will not participate in this study.

**How will my child's information be kept confidential?**

The data you give us is protected. We will collect the answers to the questions using tablets. Once the questions are collected, the answers will be locked on the tablet until they are transferred to a secure location at the CRSN and then sent to a secure server. Only study staff will be allowed to see these responses. Written consents will be kept in locked cabinets at the CRSN accessible only to study staff.

There is always a risk of loss / breach of confidentiality of study data, but we will take steps to prevent this from happening.

By signing this consent form, you authorize us to use your child's personal and medical information as described in this document.

- Your child's personal and medical information can be accessed by UCSF and others (such as regulators and ethics committees). These measures are designed to ensure that the study is conducted appropriately.
- In addition, only study staff are allowed to use information that identifies you and your child (such as your child's name) for study purposes only.
- The study information will have a code number. They will not include your child's name. The key linking the code and the child's name will be maintained by the PI or a delegate.
- The key linking your child's name and code number will not be disclosed (only coded information will be used for the study).
- CRSN and UCSF can:

o store data in electronic format and analyze for study results o Share data with authorized regulatory agencies.

o Share data with ethics committees

o share coded information with companies, organizations or universities for research purposes.

The personal and medical data collected could be transferred, stored and used in your country of residence but also in any other country where the institutions collaborate with UCSF or CRSN.

This information may be used in countries where data protection is lower than in your country of residence. UCSF and CRSN will ensure that the data transferred is processed in accordance with the consent form you signed.

The clinical trial is described on [http://www.clinicaltrials.gov,](http://www.clinicaltrials.gov/) according to American law.

**What is the cost of participating in the study?**

You and your children will not be charged for any of the study activities.

**Will my child and I be paid for participating in the study?**

You and your child will not be paid for participating in this study.

**What happens if my child is injured as a result of participating in this study?**

It is important that you inform a member of the CRSN personnel if you think that your child was injured as a result of participating in this study. You will not have to pay for medical treatment if your child is injured because of their participation in this study.

**What are the rights of my child if they participate in this study?**

The participation of your child in this study is your choice. You can choose to participate or not in the study. If you decide that your child will participate in the study, your child can quit the study at any moment. No matter what decision you make, there will be no penalty for you or your child. Leaving the study will not affect your child's medical care.

**Who can answer my questions about the study?**

If you have any questions about the study or your rights as a participant to someone other than the researchers or if you have any concerns about the study, please contact the Chair of the Health Research Ethics Committee (CERS), Prof. Seni Kouanda 09 P Boîte 7009 Ouagadougou 09, tel. +226 50 36 6674 or the President of the CRSN Institutional Ethics Committee, Mr. Zoumbara Jean Désiré, tel. +22670716530, P Box 02, Nouna.

This is a medical research study. This study will be explained to you by a staff member of the Centre de Recherche en Santé de Nouna (CRSN). Dr Ali Sié, CRSN Principal Investigator, telephone: +22670252957 is available to answer your questions, Dr Mamadou Bountogo, CRNS Coordinator, telephone: +2267030398944 and the other Principal Investigator, Dr Thomas Lietman of the University of California at San Francisco, is also available to answer your questions by telephone: +1 415-502-2662


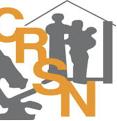

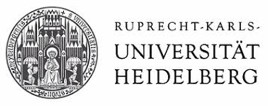

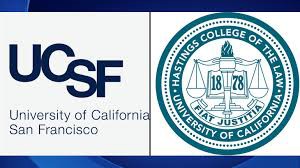


**Attest of Consent**

Participant ID:

I, the undersigned:

Name of Parent or Legal Guardian

Name of Child/Participant

I authorize my child to participate in this study.

Date Parent/ Legal Guardian or fingerprint (in case the participant is illiterate)

Date Witness Signature or fingerprint (in case the participant is illiterate)

Date Signature of the Person who obtained the Consent
