# Supplementary material for: A double-masked placebo-controlled trial of azithromycin to prevent child mortality in Burkina Faso, West Africa: Community Health with Azithromycin Trial (CHAT) study protocol
Source: Trials. 2019 Dec 4;20:675. doi: 10.1186/s13063-019-3855-9 (PMC6894235; doi:10.1186/s13063-019-3855-9)
Supplement: Supplementary file 2 — Additional file 2. Sample informed consent documents. [file 13063_2019_3855_MOESM2_ESM.zip › signed-parental-community-2.0R1.docx]

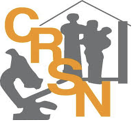

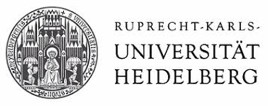

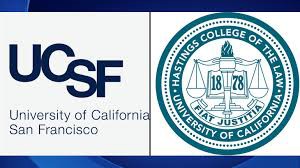


**Parental or Legal Guardian Consent for Participation in the Study**

**Information Notice:**

For parents or legal guardians of children under 5 years of age

Title: Utilization of Azithromycin for the Reduction of Mortality in Young Children Date: 10/09/2018

Version: 3.0

**Significance of Consent**:

“Consent” signifies that you authorize your child to participate in this clinical research study. You have the right to decide if you want your child to participate or not. The objective of this document is to explain the study to you. Please take the time to read or listen attentively to the following information.

This is a medical research study. This study will be explained to you by a staff member from the Centre de Recherche en Santé de Nouna (CRSN). The principal investigator, Dr. Ali Sié from the CRSN, tel: +22670252957 and the other principal investigator for this study, Dr Thomas Lietman from the university of California in San Francisco, are available by phone to answer your questions at +1 415-502-2662.

This medical research only includes the people who have chosen to participate. Take your time in making your decision to participate in this study. You can discuss your decision with your friends and family. If you have questions, you can ask the study physicians or the CRSN personnel. We ask for the participation of your child that is under five years old because your child lives in a village that is part of the study. You could choose to participate in this study or not. The head of household has already agreed to the participation of the household, but this does not mean your child has to participate. The participation of your child is voluntary. If you accept that your child participates, you will receive a copy of this signed document.

**Why is this study being conducted?**

The objective of this study to look at how the distribution of antibiotics affect childhood mortality.

**What is the role of CRSN?**

The Center of Health Research in Nouna is one of four research institutes in the ministry of health. It has functioned as a demographic health surveillance system since 1992. The DHSS of Nouna is located in the health district of Nouna in northwest Burkina Faso, about 300 kilometers from the capital, Ouagadougou. The CRSN explores various subjects depending on national and global research priorities. The CRSN has more than 20 years of professional experience within the communities of the Nouna region.

**What is the role of the University of San Francisco?**

UCSF is a public research university in San Francisco, California. For this project, UCSF will oversee all study related activities. UCSF investigators will design, analyze, and provide oversight for this research in close collaboration with the CRSN. In addition, UCSF will coordinate monitoring, training, and evaluation visits throughout the duration of the study.

**How many children/households will participate in this study?**

Approximately 20,000 households per year for 3 years will participate in this study. Approximately 50,000 children per year for 3 years will participate in this study.

**What happens if your child participates in the study?**

If you decide to participate in this study, we will come every 6 months during a 3 year period and:

-Treat your child with a dose of antibiotics (Azithromycin) or a placebo.

- The allocation of Azithromycin or placebo is randomized randomly
- If your community is randomized for Azithromycin, your child will receive Azithromycin during mass distribution
- If your community is randomized for placebo, your child will receive a placebo during mass distribution.
- Your child will receive a treatment. Treatment is a liquid solution that your child can swallow.
- Your child could receive the antibiotic, or they could receive the placebo. A computer program will randomly decide which group your child will be in, like a lottery. We cannot know or decide which group your child will be in.
- The placebo will look and taste the same as the antibiotic, but it will not contain any active substance.

-Measure the mid-upper arm circumference of your child to detect if your child is at risk of malnutrition.

-Take the GPS coordinates of your household to help us find it the next time we come

-Examine your child if your community and child is randomly picked for examination: Certain communities will be randomly chosen for an examination once a year over a 3-year period. If your community is selected for the exam, your child could be randomly selected to be examined. Randomly selected means that your child will be chosen arbitrarily by a computer program, it is like being chosen by chance, like a lottery. We cannot know in advance if your child will be chosen or not. If your child is chosen for the exam, we will:

- **Measure your child's height, weight and mid-upper arm circumference** to make sure your child is not at risk for malnutrition
- **Take a photo of your child’s face**. This will only be done to identify your child if we examine them more than once.
- **Take a blood sample from your child’s finger or heel**: a qualified medical worker will prick your child’s finger or heel to obtain a drop of blood (approximately 0.2ml). We will test the blood for malaria and anemia. If your child is anemic, we will refer you to the nearest CSPS or to the CMA in Nouna.
- **Take a nasal sample from your child**: a trained medical officer will insert a flexible swab with a cotton tip in your child’s nose until it reaches the back of the nose called nasopharynx.
- **Obtain a stool sample from your child**: 2 methods are used to obtain a fecal sample from your child. Either a qualified health professional will insert a flexible swab with a cotton tip in the buttocks of your child to obtain a rectal sample, or your child will have to discharge feces in a plastic bag and a trained health professional will take a sample of that.

**What will become of the samples taken during the study?**

The samples taken from your child will be frozen and stored without your child’s name or any other identification at the CRSN in Nouna and in the Heinz Laboratory at the F.I. Proctor Foundation at 513 Parnassus Avenue, S310, San Francisco, CA 94143.

If you agree to let us collect and store your child's specimen for future research, after all tests for this study are performed, we will keep them for 30 years in what is called a "tissue bank” for future research. If you subsequently decide that you do not want your child's samples and information used for future research, you may notify us by calling the CRSN in Nouna at +22670252957, and we will destroy all remaining identifiable samples and information. However, if research has already been done on parts of your specimens, the data will be retained and analyzed as part of this research.

**How long will my child be in the study?**

Each visit to treat your child will take approximately 15 minutes. We will visit your child 7 times over a period of 3 years (105 minutes total).

The visit for the exam will take approximately 30 minutes once a year if your community and child are randomly selected: 5 minutes for the anthropometry measurements, 10 minutes for the blood sample, and 10 minutes for the nasopharyngeal and rectal samples.

**Can my child stop participating in the study?**

Yes. You can decide to stop participating at any moment. Inform the study physician or the CRSN personnel if you are considering stopping or if you decide to stop. They will tell you how to stop your child’s participation safely.

**What are the secondary effects or the risks my children can expect to suffer?**

Your child can have secondary effects during the study. The secondary effects frequently associated with this type of antibiotic are: diarrhea, abdominal pain, vomiting, or skin rashes. Each child participating in this study will be carefully monitored for any side effects.

Your child may also be allergic to the study medication, the most common allergic reactions to this type of treatment are: a rash, itching or dizziness. Serious allergic reactions such as shortness of breath or swelling of the face / tongue / lips are rare and will be closely monitored and treated free of charge by our team.

The antibiotic treatment can change the variability of the types of bacteria in your child’s gut. Some research suggests that this change will only be temporary and the diversity of bacteria living in your child’ s intestine and nose will return to normal after a few weeks after treatment. However, it is not guaranteed that this return to normalcy of bacteria will occur.

Risk of injury during the exam procedure is minimal. Your child may experience discomfort when we collect the blood sample, but the risk of injury is minimal. Nose bleeding can occur after nasopharyngeal swabs, if this is the case, a medical staff member will treat your child immediately. You should talk to a CRSN staff member if your child is experiencing any side effects.

**What are the advantages of participating in the study?**

If your child is in the group that receives the antibiotic, they may benefit from participating in the study if they suffer from undiagnosed disease, but this cannot be guaranteed.

**What other choices do I have if my child is not participating in this study?**

You can decide to participate in the study or not to participate in the study. Your participation is voluntary. It is your choice if your child participates in the study or not. Nothing will happen to you or your child if you decide that your child will not participate in this study.

**How will my child's information be kept confidential?**

The data you give us is protected. We will collect the answers to the questions using tablets. Once the questions are collected, the answers will be locked on the tablet until they are transferred to a secure location at the CRSN and then sent to a secure server. Only study staff will be allowed to see these responses. Written consents will be kept in locked cabinets at the CRSN accessible only to study staff.

There is always a risk of loss / breach of confidentiality of study data, but we will take steps to prevent this from happening.

By signing this consent form, you authorize us to use your child's personal and medical information as described in this document.

- Your child's personal and medical information can be accessed by UCSF and others (such as regulators and ethics committees). These measures are designed to ensure that the study is conducted appropriately.
- In addition, only study staff are allowed to use information that identifies you and your child (such as your child's name) for study purposes only.
- The study information will have a code number. They will not include your child's name. The key linking the code and the child's name will be maintained by the PI or a delegate.
- The key linking your child's name and code number will not be disclosed (only coded information will be used for the study).
- CRSN and UCSF can:

o store data in electronic format and analyze for study results o Share data with authorized regulatory agencies.

o Share data with ethics committees

o share coded information with companies, organizations or universities for research purposes.

The personal and medical data collected could be transferred, stored and used in your country of residence but also in any other country where the institutions collaborate with UCSF or CRSN.

This information may be used in countries where data protection is lower than in your country of residence. UCSF and CRSN will ensure that the data transferred is processed in accordance with the consent form you signed.

The clinical trial is described on [http://www.clinicaltrials.gov,](http://www.clinicaltrials.gov/) according to American law.

**What is the cost of participating in the study?**

You and your children will not be charged for any of the study activities.

**Will my child and I be paid for participating in the study?**

You and your child will not be paid for participating in this study.

**What happens if my child is injured as a result of participating in this study?**

It is important that you inform a member of the CRSN personnel if you think that your child was injured as a result of participating in this study. You will not have to pay for medical treatment if your child is injured because of their participation in this study.

**What are the rights of my child if they participate in this study?**

The participation of your child in this study is your choice. You can choose to participate or not in the study. If you decide that your child will participate in the study, your child can quit the study at any moment. No matter what decision you make, there will be no penalty for you or your child. Leaving the study will not affect your child's medical care.

**Who can answer my questions about the study?**

If you have questions about the study or your rights as a participant to someone other than the researchers or if you have concerns about the study, please contact the President of the Ethics for Health Research Committee Professor Seni Kouada 09 P Box 7009 Ouagadougou 09, telephone: +226 50 36 6674 or the President of the CRSN Ethical Committee: M Zoumbara Jean Desire, telephone: +22670716530, P Box 02, Nouna

This is a medical research study. This study will be explained to you by a member of the CRSN personnel. Dr. Ali Sie, Director of CRSN, telephone: +22670252957 is here to answer your questions. Dr. Mamadou Bountogo, the coordinator of the study at CRSN, telephone: +22670398944 and the other Principal Investigator, Dr. Thomas Lietman at the University of California San Francisco is available to answer your questions by telephone at +1 415-502-2662.


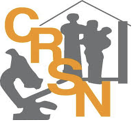

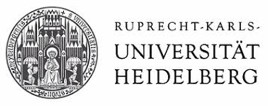

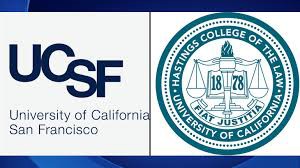


**Attest of Consent**

Participant ID:

I, the undersigned:

Name of Parent or Legal Guardian

Name of Child/Participant

I have read the informed consent form, or it was read to me. I understand perfectly the information here above. I had the opportunity to ask questions, and I had satisfactory answers. I voluntarily consent to have my child participate in this research. I understand that I have the right to refuse my participation or to stop at any moment without penalty or loss of benefits which I am otherwise entitled.

Check the corresponding box.

I agree that the samples from my child will be used for further study. These samples will be coded, but de-identified to my child’s identity.

Yes🞏 No🞏

I authorize my child to participate in this study.

Date Parent/ Legal Guardian or fingerprint (in case the participant is illiterate)

Date Witness Signature or fingerprint (in case the participant is illiterate)

Date Signature of the Person who obtained the Consent
